# Supplementary material for: Agronomic Investigation of Spray Dispersion of Metal-Based Nanoparticles on Sunflowers in Real-World Environments
Source: Plants (Basel). 2023 Apr 27;12(9):1789. doi: 10.3390/plants12091789 (PMC10180907; doi:10.3390/plants12091789)
Supplement: Supplementary file 1 [file plants-12-01789-s001.zip › plants-2288204-supplementary.pdf]

plants

Supplementary Materials

Agronomic investigation of spray-dispersion of metal-based nanoparticles on sunflower in real-world environments

Table S1. X-ray diffraction analysis of magnetite (Fe_3O_4) with related crystal symmetry dimension and unit cell parameters.

	Magnetite crystallographic determination
Crystal Symmetry	cubic
a-axes	8.3905 ± 0.0001 Å
α, α, α	90°
Space Group	*Fd*-3*m*
Unit Cell Volume *	590.695 Å^3
Lvol-IB (Nanometers) **	5.4 ± 0.2 nm

Note: * calculated from Unit Cell, ** Calculated from X-ray diffraction analysis.

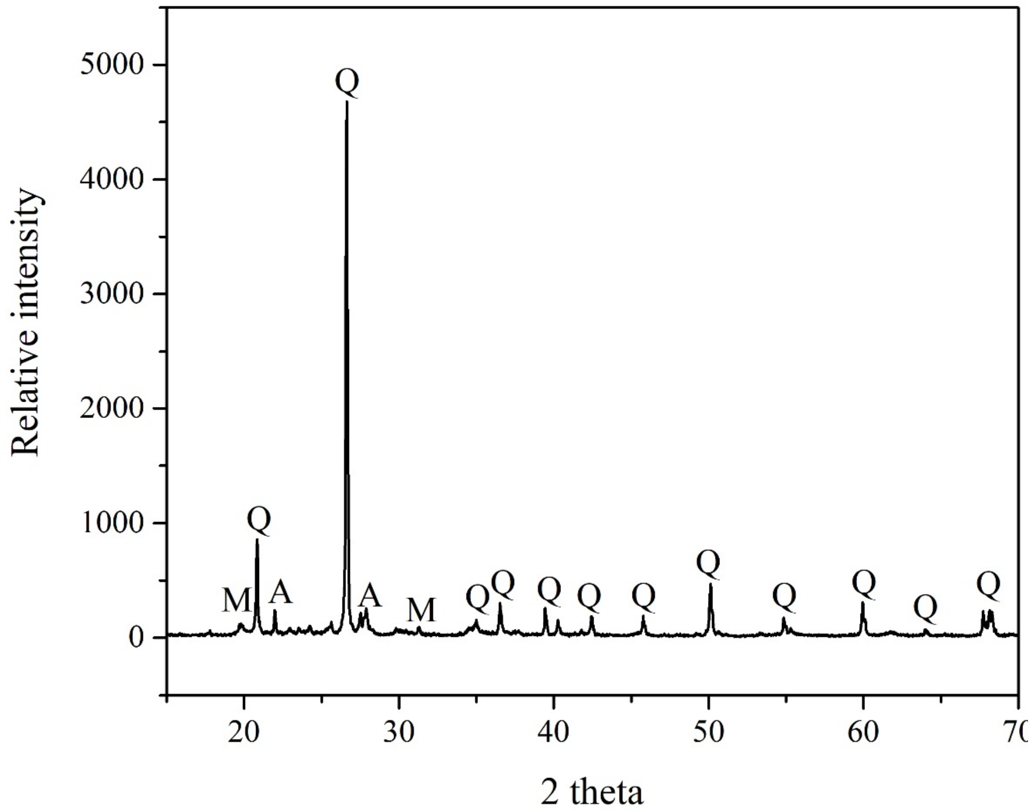

Figure S1. X-ray diffraction analysis of soil samples of A-horizons from Dolná Malanta in Nitra, Slovakia, Central Europe shown typical minerals such as **Q**uartz (SiO2), **M**uscovite ($KAl_2(Si_3Al)O_{10}(OH,F)_2$) and **A**nortite ($NaAlSi_3O_8$).

Table S2. Trichome width (± standard deviation) found on leaves which were collected during flower bud formation after two foliar applications of AuSi-NPs, Fe_3O_4-NPs and ZnO-NPs and the NP-free control treatment.

Type of Trichomes	Leaf Side	Control	AuSi-NPs	Fe_3O_4-NPs	ZnO-NPs
NGTs					
Mean (µm)	AD	86.36 ± 17.78a	77.88 ± 19.53b	82.52 ± 22.27ab	84.56 ± 20.02a
	AB	52.61 ± 14.74a	54.79 ± 14.31ab	58.14 ± 18.17b	53.80 ± 15.96ab
Min (µm)	AD	52.2	36.3	37.2	43.5
	AB	26.6	30.6	20.4	26.9
Max (µm)	AD	152.0	150.7	159.8	157.6
	AB	116.7	108.7	130.5	117.1
C_v (%)	AD	20.6	25.1	27.0	23.7
	AB	28.0	26.1	31.3	29.7
LGTs					
Mean (µm)	AD	67.05± 7.85a	65.55 ±7.79ab	63.50 ± 8.73b	65.43 ± 8.97ab
	AB	57.98 ± 7.84a	60.28 ± 8.28a	58.71 ± 7.60a	58.82 ± 7.52a
Min (µm)	AD	46.4	51.1	46.5	44.9
	AB	41.1	45.4	44.5	46.8
Max (µm)	AD	92.5	91.1	102.8	92.5
	AB	80.5	92.3	82.9	84.8
C_v (%)	AD	11.7	11.9	13.7	13.7
	AB	13.5	13.7	12.9	12.8
CGTs					
Mean (µm)	AD	-	-	-	-
	AB	55.72 ±3.36a	55.33 ± 3.49a	56.57 ± 3.95ab	57.63 ± 3.45b
Min (µm)	AD	-	-	-	-
	AB	48.1	41.0	45.3	48.1
Max (µm)	AD	-	-	-	-
	AB	63.6	66.3	69.7	65.3
C_v (%)	AD	-	-	-	-
	AB	6.04	6.31	6.99	5.98

Note: AD – adaxial, AB – abaxial, Min—minimum, Max—maximum, C_v—coefficient of variation, NGTs – non-glandular trichomes, LGTs – linear glandular trichomes, CGTs – capitate glandular trichomes. Superscript letters indicate the results of Tukey's HSD test ($\alpha = 0.01$).

Table S3. Mean length and width of stomata, length and width of stomatal pores, number of stomata (N) per 100 000 μm^2 and 1 mm^2 along with standard deviation (SD), coefficient of variation (CV), minimal and maximal values (Min, Max).

	**Control**	**AuSi-NPs**	**Fe_3O_4-NPs**	**ZnO-NPs**
Length of Stomata				
Mean ± SD (μm)	30.10 ± 4.00^{b}	28.67 ± 4.30^{a}	29.22 ± 4.61^{ab}	29.36 ± 4.94^{ab}
CV (%)	13.31	14.99	15.79	16.84
Min (μm)	19.61	17.52	16.92	16.98
Max (μm)	40.51	39.85	41.38	41.25
Width of Stomata				
Mean ± SD (μm)	18.99 ± 3.17^{a}	18.84 ± 3.06^{a}	17.90 ± 3.88^{b}	18.59 ± 3.70^{ab}
CV (%)	16.69	16.23	21.65	19.93
Min (μm)	10.03	10.71	9.32	10.42
Max (μm)	27.15	29.17	29.38	28.90
Length of Stomatal Pores				
Mean ± SD (μm)	18.30 ± 3.15^{ab}	18.28 ± 3.24^{b}	17.69 ± 3.14^{a}	17.74 ± 3.44^{ab}
CV (%)	17.19	17.74	17.73	19.38
Min (μm)	9.68	8.45	9.80	9.43
Max (μm)	27.08	33.86	28.49	28.08
Width of Stomatal Pores				
Mean ± SD (μm)	3.19 ± 0.96^{b}	2.82 ± 0.93^{a}	2.82 ± 1.03^{a}	2.88 ± 0.78^{a}
CV (%)	30.14	33.09	36.41	27.02
Min (μm)	0.87	0.88	1.03	1.11
Max (μm)	7.13	5.89	6.16	5.79
Number of Stomata per 100 000 μm^2				
Mean ± SD (pcs)	25.03 ± 5.38^{a}	21.00 ± 6.67^{ab}	20.08 ± 5.38^{b}	25.00 ± 7.30^{a}
CV (%)	21.48	31.75	26.80	29.20
Min (pcs)	15	5	9	7
Max (pcs)	41	38	36	42
Number of Stomata per 1 mm^2				
Mean (pcs)	250.25	210.00	200.75	250.00

Note: The values present after symbol ± show the variance of the values as standard deviation. Superscript letters indicate the results of Tukey's HSD test (α = 0.01).

Table S4. Abundance and dominance of the family Coleoptera in the studied treatments with common sunflower during vegetation season 2019.

Family	Control	AuSi-NPs	ZnO-NPs	Fe_3O_4-NPs	Σ	Dominance (%)
Anthicidae	14	9	3	13	39	1.85
Carabidae	720	747	254	244	1 965	93.44
Cerambicidae	ND	ND	1	2	3	0.14
Coccinelidae	ND	6	ND	5	11	0.52
Curculionidae	3	ND	2	ND	5	0.24
Dermestidae	8	11	3	4	26	1.24
Elateridae	ND	12	ND	10	22	1.05
Histeridae	ND	ND	1	ND	1	0.05
Silphidae	7	ND	ND	4	11	0.52
Staphylinidae	5	4	2	ND	11	0.52
Tenebrioidae	9	ND	ND	ND	9	0.43
Σ	766	789	266	282	2 103	100.00

Note: * ND – not detected.

Table S5. Abundance and dominance of the Carabidae species in the studied treatments with common sunflower during vegetation season of 2019.

Species	Control	AuSi-NPs	ZnO-NPs	Fe_3O_4-NPs	Σ	Dominance (%)
Anchomenus dorsalis	8	1	2	5	16	0.81
Brachinus crepitans	70	75	28	22	195	9.92
Brachinus explodens	7	ND	ND	6	13	0.66
Calosoma auropunctatum	ND	ND	ND	3	3	0.15
Carabus scheidleri	ND	ND	ND	5	5	0.25
Dolichus halensis	6	ND	1	3	10	0.53
Harpalus flavescens	ND	ND	ND	9	9	0.45
Chlaenius festivus	4	ND	ND	ND	4	0.22
Poecilus cupreus	ND	6	74	6	86	4.37
Harpalus rufipes	614	663	148	185	1 610	81.93
Zabrus tenebrioides	11	2	1	ND	14	0.71
Σ	720	747	254	244	1 965	100.00

Note: * ND – not detected.
